# A correction to the age-adjustment of the GH-2000 score used in the detection of growth hormone misuse

**DOI:** 10.1186/s13104-018-3741-7

**Published:** 2018-09-05

**Authors:** Dankmar Böhning, Walailuck Böhning, Nishan Guha, David A. Cowan, Christiaan Bartlett, Peter H. Sönksen, Richard I. G. Holt

**Affiliations:** 1 Southampton Statistical Sciences Research Institute, University of Southampton, Southampton, SO17 1BJ UK; 2 Human Development and Health Academic Unit, Faculty of Medicine, Southampton General Hospital, University of Southampton, Southampton, SO16 6YD UK; 3 Nuffield Division of Clinical Laboratory Sciences, UK Department of Clinical Biochemistry Level 4, University of Oxford, John Radcliffe Hospital Headley Way, Headington, Oxford, OX3 9DU UK; 4 Department of Pharmacy and Forensic Science, Drug Control Centre, King’s College London, 150 Stamford Street, London, SE1 9NH UK

**Keywords:** GH-2000 score, Adjusting for age effects, Centring and norming of scores

## Abstract

**Objective:**

The GH-2000 biomarker test has been introduced by the World Anti-Doping Agency as a method of detecting growth hormone misuse in professional sport. The test involves the measurement insulin-like growth factor-I and the amino-terminal pro-peptide of type III collagen (P-III-NP) which increase in a dose-dependent manner in response to GH. These measurements are combined in sex specific formulae that include an age adjustment. The original age adjustment overcorrects the effect of age in male athletes and could potentially place older men at a disadvantage. The purpose of this note is to investigate the performance of a previously suggested correction term in two new and larger data sets.

**Results:**

The GH-2000 score was calculated for 7307 samples obtained from 15 accredited WADA laboratories in 2017 and 3916 samples measured at Drug Control Centre, King’s College London, UK between 2013 and 2017. The GH-2000 scores were investigated for positive age effects using standard regression modelling. As previously, all analyses confirmed a positive age effect. Applying the earlier suggested correction term of 0.032 × age showed a significant over-correction leading to a negative association of the GH-2000 score with age. We now suggest a smaller age correction of 0.020 × age, which corresponds to the smallest effect found in the earlier studies.

**Electronic supplementary material:**

The online version of this article (10.1186/s13104-018-3741-7) contains supplementary material, which is available to authorized users.

## Introduction

Growth hormone is not only a powerful anabolic agent of considerable therapeutic value but is also misused in sport for its anabolic and lipolytic properties and its use is banned by the World anti-doping agency [1–3].

The GH-2000 biomarker method to detect GH misuse is based on the measurement of the GH-sensitive biomarkers, insulin-like growth factor-I (IGF-I) and the amino-terminal pro-peptide of type III collagen (P-III-NP), both of which rise following GH administration. The GH-2000 score was initially developed using immunoassays that are no longer available [4, 5]. Although the original discriminant function has remained unchanged, the decision limits have been updated as further experience has been accumulated and new assays have become available [6].

Currently, there are three IGF-I assays (LC–MS/MS, Immunotech, IDS) and two P-III-NP (ORION and Siemens-Centaur) assays approved by WADA. For more details and background on these assays see [6]. As these assays do not give identical results, different GH-2000 scores are obtained with each of the combinations and as a result the decision limits are different, depending on the assay pair used.

The GH-2000 score includes an age correction because GH secretion and its markers diminish with age; in the case of GH, its secretion falls by ~ 14% per decade. The original age correction was determined using samples from 813 elite athletes; however, we previously reported that when using the original correction, the GH-2000 score showed a positive age effect in male athletes competing at the Daegu World Athletics Championship in 2011. We proposed a correction term for inclusion in the GH-2000 score [7].

The aim of the current study is assess the effect of the original and updated age correction in two new large populations of elite male athletes.

## Main text

### The GH-2000 score

The development of the GH-2000 score has been reported previously in [6, 7]. It has the theoretical or model form

1 $${\text{GH-2000 score}}\, = \,\beta_{0} \, + \,\beta_{1} { \log }\left( {\text{IGF-I}} \right)\, + \,\beta_{2} { \log }\left( {\text{P-III-NP}} \right)\, + \,{{\beta_{3} } \mathord{\left/ {\vphantom {{\beta_{3} } {\text{age}}}} \right. \kern-0pt} {\text{age}}}$$

where the coefficients $\beta_{0} ,\beta_{1} ,\beta_{2} ,\beta_{3}$ have different values for male and female athletes. When coefficients are replaced by estimates the GH-2000 score for male athletes is

2 $${\text{GH-2000 score}}\, = \, - \,6.586\, + \,2.100{\ \log }\left( {\text{IGF-I}} \right)\, + \,2.905{\ \log }\left( {\text{P-III-NP}} \right)\, - \,{{101.737} \mathord{\left/ {\vphantom {{101.737} {\text{age}}}} \right. \kern-0pt} {\text{age}}}$$

and for female athletes

$${\text{GH-2000 score}}\, = \, - \,8.459\, + \,2.195{\ \log }\left( {\text{IGF-I}} \right)\, + \,2.454{\ \log }\left( {\text{P-III-NP}} \right)\, - \,{{ 7 3. 6 6 6} \mathord{\left/ {\vphantom {{ 7 3. 6 6 6} {\text{age}}}} \right. \kern-0pt} {\text{age}}}$$

As the GH-2000 score showed positive age-dependency for male athletes competing at the Daegu World Athletics Championship in 2011, an adjustment to age correction was suggested in [5] as follows:

3 $$\begin{aligned} {\text{GH-2000 score}}\, = & \, - \,6.586\, + \,2.100{ \ \log }\left( {\text{IGF-I}} \right)\, + \,2.905{\ \log }\left( {\text{P-III-NP}} \right) \\ \, & {{ - \,101.737} \mathord{\left/ {\vphantom {{ - \,101.737} {\text{age}}}} \right. \kern-0pt} {\text{age}}}\, - \, 0. 0 3 2 {\text{ (age}}\, - \, 2 5. 0 9 )\\ \end{aligned}$$

This correction is only required for the male population of athletes as no age dependency was found for female athletes. When applying the corrected score (3) to the GH-2000 score data used in [5] the positive age effect was no longer seen.

We have investigated two larger data sets, which includes data on testing for growth hormone misuse using the GH-2000 methodology. The first one contains results of serum IGF-I and P-III-NP and calculated GH-2000 score from 7307 analyses in men performed in 15 accredited WADA laboratories. WADA made these data available to the GH-2004 team. We denote this data set as the 2017-WADA. The second data set stems from the Drug Control Centre, King’s College London, UK and contains 3916 analyses in men collected in the years 2013–2017. We denote this second data set as the London Lab 2012–2017 data. These are the largest current databases on GH-2000 scores available.

## Results

**Fig. 1:**
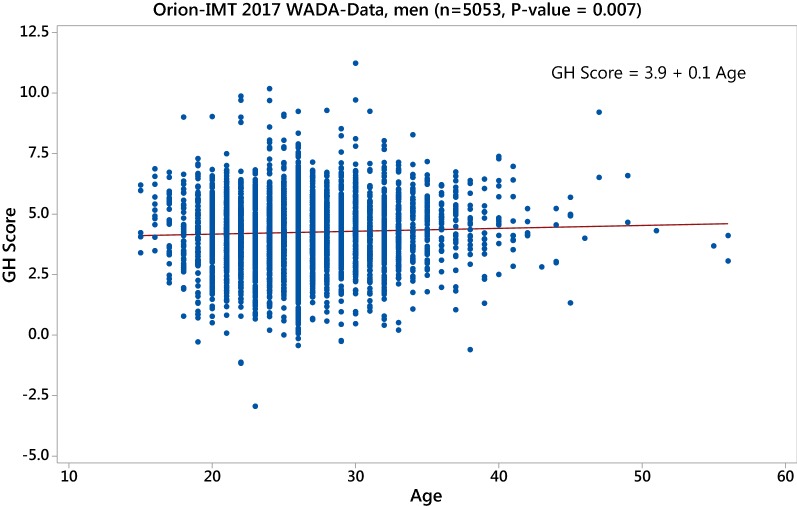
Uncorrected GH-2000 score (2) using the assay combination Immunotech and ORION in the 2017 WADA data set showing a significant positive age effect

**Fig. 2:**
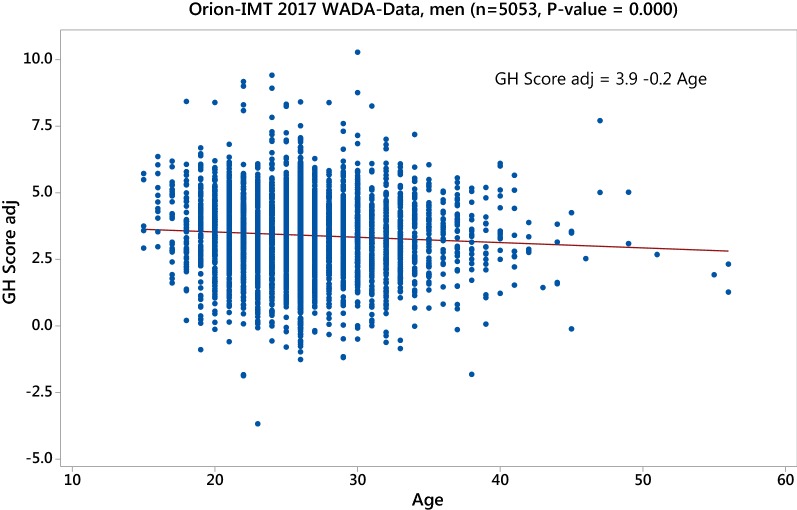
Corrected GH-2000 score (3) using the assay combination Immunotech and ORION in the 2017 WADA data set showing a significant negative age effect

Both available data sets were used to investigate any age effect in the GH-2000 score. Although different pairs of assays were used, the combination of the Immunotech-ORION assays was most frequently used. In Fig. 1 and Figure S1 of Additional file 1, the positive age dependency is clearly visible when the original GH-2000 score based on the combination of the Immunotech-ORION (2) is used. This is also true when the LC–MS/MS and Siemens assay combination is used in the 2017 WADA data set (Figure S4 of Additional file 1, panel a). When the additional correction term (3) is applied, we observed a significant negative age-affect in Fig. 2 and Figure S2, S4b of Additional file 1.

As the additional correction term (3) leads to over-correction of the effect of age in both datasets, we have reconsidered the correction term 0.032 used in (3). It was developed as weighted average of the age-effects across the various assay combinations. The specific age-effect for the Immunotech-ORION combination in Table 1 from [7] is 0.0202. We therefore propose using this value as the correction term as it is represents the Immunotech-ORION specific age-effect. The adjusted GH-2000 score then becomes:

4 $$\begin{aligned} {\text{GH-2000 score}}\, = & \, - \,6.586\, + \,2.100{ \ \log }\left( {\text{IGF-I}} \right)\, + \,2.905{ \ \log }\left( {\text{P-III-NP}} \right) \\ \, & - \,{{101.737} \mathord{\left/ {\vphantom {{101.737} {\text{age}}}} \right. \kern-0pt} {\text{age}}}\, - \, 0. 0 2 {\text{ (age}}\, - \, 2 5. 0 9 )\\ \end{aligned}$$

**Fig. 3:**
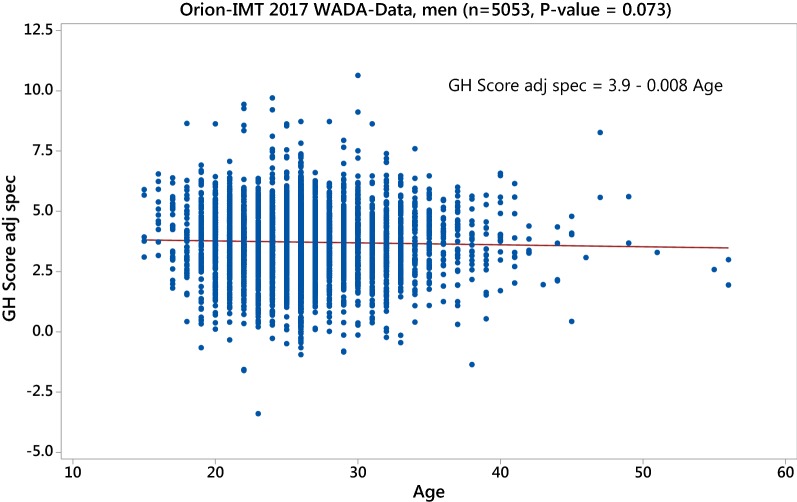
GH-2000 score with new correction term (4) using the assay combination Immunotech and ORION in the 2017 WADA data set showing a *non*-*significant* age effect

If we apply this correction for the Immunotech-ORION assay combination in the 2017 WADA and the London Lab 2012–2017 data set, we see that the age-effect disappears as illustrated in Fig. 3.

The age-correction of 0.020 which is implemented in the new adjusted GH-2000 score also works well for other assay combinations as Figures S3 and S5 of Additional file 1 show where (4) is applied to the LC–MS/MS and Siemens assay combination. It should be noted from Table 1 in [7] that 0.020 is also the smallest observed age-affect. Hence we recommend this value instead of the previously suggested value of 0.032.

## Discussion

We are proposing this revised age adjustment for only the male athlete population as the original age adjustment performed appropriately for the female population in these new cohorts of athletes. The original GH-2000 discriminant function for women therefore remains unchanged.

It is clear that the age-effect correction suggested in [7] over-corrects and can introduce a negative age-affect, hence favouring older athletes in these two larger populations. We have demonstrated that our new proposed age-effect correction removes the positive age effect in the two newer datasets as well as the Daegu World Athletics Championship data set used in [7].

This new correction term of 0.02 is consistent with the data set used in [7] as it is the specific term found for the Immunotech-ORION assay combination. Taken together with the correction of the age effect in the two new independent datasets, this provides strong evidence through confirmation of the validity of the findings. It is important to recognise that we are not using new estimates from the new two data sets, but are using the estimates of the age-effect found in [7].

## Limitations

This newly proposed adjustment term needs to be monitored in future data sets, either as part of routine monitoring, as in this case, or as part of designed studies.

In conclusion, we have created a small further age adjustment for male athletes to correct the age bias introduced with the original discriminant formula. This has no effect on the decision limits and could be easily introduced into anti-doping testing.

## Additional file

**Additional file 1.** Additional figures.
