# Supplementary material for: A correction to the age-adjustment of the GH-2000 score used in the detection of growth hormone misuse
Source: BMC Res Notes. 2018 Sep 5;11:650. doi: 10.1186/s13104-018-3741-7 (PMC6125940; doi:10.1186/s13104-018-3741-7)
Supplement: Supplementary file 1 — Additional file 1. Additional figures. [file 13104_2018_3741_MOESM1_ESM.docx]

***Additional file***

**A Correction on the Age-Adjustment of the GH-2000 Score Detecting Growth Hormone Misuse**

Dankmar **Böhning**^[[1]](#footnote-1)^**^*^**, Walailuck **Böhning**^2^, Nishan **Guha**^2,3^ David A**. Cowan**^4^, Christiaan **Bartlett**^4^, Peter H. **Sönksen**^2^ and Richard I.G**. Holt**^2^

**Figure S1:**  uncorrected GH-2000 score (2) using the assay combination Immunotech and ORION in the London Lab 2012-2017 data set showing a significant positive age effect

**Figure S2:** corrected GH-2000 score (3) using the assay combination Immunotech and ORION in the London Lab 2012-2017 data set; showing a significant negative age effect

**Figure S3:** GH-2000 score with new correction term (4) using the assay combination Immunotech and ORION in the London Lab 2012-2017 data set showing a *non-significant* age effect

a)

b)

**Figure S4:** GH-2000 score using the assay combination LC-MS/MS and Siemens in the 2017 WADA data set; a) uncorrected GH-2000 score (2) showing a significant positive age effect, b) corrected GH-2000 score (3) showing a significant negative age effect

**Figure S5:** GH-2000 score with new correction term (4) using the assay combination LC-MS/MS and Siemens in the 2017 WADA 2012-2017 data set showing a *non-significant* age effect

1. *Correspondence: d.a.bohning@soton.ac.uk

   Southampton Statistical Sciences Research Institute, University of Southampton, Southampton, SO17 1BJ, UK [↑](#footnote-ref-1)
